# Blood lactate levels in patients receiving first- or second- generation antipsychotics

**DOI:** 10.3325/cmj.2011.52.41

**Published:** 2011-02

**Authors:** Trpimir Glavina, Damir Mrass, Tajana Dodig, Gordana Glavina, Shelly Pranić, Boran Uglešić

**Affiliations:** 1 Psychiatric Clinic, Split Clinical Hospital Center, Split, Croatia; 2 Vrapče Psychiatric Hospital, Zagreb, Croatia; 3 Clinical Institute of Radiology, Split Clinical Hospital Center, Split, Croatia; 4 University of Split School of Medicine, Split, Croatia

## Abstract

**Aim:**

To compare the blood lactate levels between patients with psychotic disorder receiving first- and those receiving second-generation antipsychotics.

**Methods:**

The study was conducted at the psychiatric inpatient and outpatient clinics of the Split Clinical Hospital from June 6, 2008 to October 10, 2009. Sixty patients with psychotic disorder who were assigned to 6-month treatment were divided in two groups: 30 received haloperidol (first generation antipsychotic) and 30 received olanzapine (second generation antipsychotic). Blood lactate levels, other metabolic parameters, and scores on the extrapyramidal symptom rating scale were assessed.

**Results:**

Patients receiving haloperidol had significantly higher blood lactate levels than patients receiving olanzapine (*P* < 0.001). They also more frequently had parkinsonism, which was significantly correlated with both haloperidol treatment at 1 month (*P* < 0.001) and 6 months (*P* = 0.016) and olanzapine treatment at baseline (*P* = 0.016), 3 months (*P* = 0.019), and 6 months (*P* = 0.021). Also, patients receiving haloperidol had significant correlation between blood lactate and dystonia at 1 month (*P* < 0.001) and 6 months (*P* = 0.012) and tardive dyskinesia at 1 month (*P* = 0.032). There was a significant difference between the treatment groups in lactate levels at all points from baseline to month 6 (*P* < 0.001).

**Conclusion:**

It is important to be aware of the potential effect of haloperidol treatment on increase in blood lactate levels and occurrence of extrapyramidal side effects. Therefore, alternative antipsychotics should be prescribed with lower risk of adverse side effects.

**Trial identification number:**

NCT01139463

Due to their heterogeneity, antipsychotics are difficult to classify, but they are frequently categorized as the first- and second-generation based on the incidence of extrapyramidal side effects, ie, antidopaminergic activity (1,2). First-generation antipsychotics have dominant antidopaminergic activity and pronounced extrapyramidal side effects (1), while second-generation antipsychotics have a pronounced effect on other neurotransmitter systems, as well as sporadic extrapyramidal side effects.

Antipsychotics block numerous neurotransmitter receptors in a manner that induces therapeutic effects and side effects, which may vary in intensity and produce serious consequences (3-7). Extrapyramidal side effects (adverse cardiovascular, hematological, gastrointestinal, sexual, and urologic effects) are most frequently manifested in first-generation antipsychotics due to their non-selective dopaminergic block (1,8-10). The consequence of a dopaminergic effect on the tuberoinfundibular system causing dopamine to inhibit prolactin secretion is hyperprolactinemia (11,12), with possible consequences such as tissue hypoxia and mortality (13-15).

Particular attention today is paid to the effects of first-generation antipsychotics on metabolic disorders. Numerous studies have shown that first-generation antipsychotic therapy may lead to metabolic changes, particularly changes in the regulation of glucose, lipid levels, and body weight (3-5,13-21). These side effects are associated with increased mortality and substantial morbidity including diabetes, hypertension, and cardiovascular disease (22,23). In many years of clinical practice, we have empirically observed that treatment with certain antipsychotics causes, along with recognized and described metabolic disorders, an increase in the blood lactate levels. Increased lactate levels are generally associated with increased morbidity and mortality in patients with chronic illnesses or critically ill patients (13,14,24-26). A review of the literature did not find any studies on the effect of antipsychotic therapy on lactate levels or such changes as a part of other antipsychotic side effects. Therefore, it is important to investigate this phenomenon in patients taking first- or second-generation antipsychotic medication.

We hypothesized that a 6-month treatment with haloperidol or olanzapine would change blood lactate levels and cause extrapyramidal side effects in patients without prior antipsychotic treatment.

## Methods

### Participants

Men aged 20-50 years with psychotic relapse or newly diagnosed psychotic disorder who were not taking any medications apart from the prescribed antipsychotic in the period of one month before the study were recruited from psychiatric inpatient and outpatient clinics of the Split Clinical Hospital. Exclusion criteria were female sex, baseline lactate levels over 2.0 mmol/L, tobacco use, and previously diagnosed diabetes mellitus. Women were excluded due to significant oscillations in plasma lactate levels during menstruation (27-30). Psychiatric diagnoses and past treatment history were evaluated by chart review, interview with treatment clinicians, and clinical interview with the patient and/or caregiver in accordance with the International Classification of Diseases ICD-10 criteria. Each participant had previously been informed of the outline of the study and gave a signed the informed consent. This study was approved by the Ethics Committee of the University of Split School of Medicine.

### Study design

A non-randomized, prospective, assessor-blind design was used. One group of 30 participants was given haloperidol (first-generation antipsychotic) and another group of 30 participants was given olanzapine (second-generation antipsychotic). Similar studies (31,32) have used fewer than 40 participants, but we applied a more conservative approach and used 60 participants. The study lasted from June 6, 2008 to October 10, 2009. The dose of all medications remained unchanged during the study period.

### Assessments

Baseline assessments included demographics and metabolic parameters. The extrapyramidal symptom rating scale (ESRS) was used to investigate four types of antipsychotic-induced movement disorders: parkinsonism, akathisia, dystonia, and tardive dyskinesia (33). The independent examiner who evaluated extrapyramidal side effects was blind to the patients’ therapy assignment.

Laboratory assessment at baseline included lactate, fasting glucose, blood pressure, and weight. Follow-up visits occurred at 1, 3, and 6 months after the study initiation. At each follow-up visit, all baseline evaluations were repeated. Capillary blood was collected from the finger pad by a sterile lancet and capillary tube and analyzed at the Split Clinical Hospital Central Laboratory.

The primary outcome included the changes in lactate levels. In a healthy adult, the reference values of blood lactate level are <1.5 mmol/L to 10-15 mmol/L during exertion. In patients suffering from critical illness, moderate increase in blood lactate levels can range from 2-4.0 mmol/L, whereas levels >4.0 mmol/L are critically high. Lactate levels in patients suffering from metabolic diseases can range from 5-15 mmol/L (34). The major secondary outcome were changes in ESRS scores.

### Statistical analysis

All analyses were performed using the SPSS, version 13.0 (SPSS Inc., Chicago, IL, USA). Continuous variables were presented using summary statistics such as means and standard deviations. Categorical variables were presented using frequencies and percentages. Baseline characteristics were compared using independent samples *t* test. Antipsychotic treatment type and blood lactate levels from baseline to endpoint were compared by two-way repeated measures ANOVA to test the significance of mean differences, followed by a series of post-hoc pairwise *t* tests and Bonferroni correction to test the probability at which to accept any of these tests. Therefore, we accepted pairwise *t* tests as being significant only if they were *P* < 0.0167. Greenhouse-Geisser corrections were made when the assumption of sphericity was violated (35). The relationship between blood lactate level and ESRS changes was examined using Spearman correlation. Differences were considered significant at *P* < 0.05.

## Results

### Demographic and basic descriptive data

The two treatment groups did not differ significantly in age or baseline glucose, weight, and systolic and diastolic blood pressure. However, haloperidol treatment group had higher baseline lactate levels than olanzapine group (*P* = 0.045, Table 1).

**Table 1:** Baseline characteristics among 60 patients taking first generation (haloperidol) or second generation (olanzapine) antipsychotics

	Patients receiving treatment with (mean ± standard deviation)			
	haloperidol (n = 30)	olanzapine (n = 30)	*t*	*P*
Age	34.2 ± 9.8	31.1 ± 8.4	1.304	0.197
Blood lactate (mmol/L)	1.4 ± 0.4	1.2 ± 0.4	2.048	0.045
Weight (kg)	86.7 ± 11.5	85.3 ± 9.9	0.481	0.632
Glucose level (mg/dL)	5.3 ± 0.6	5.2 ± 0.6	0.625	0.535
Systolic blood pressure (mmHg)	131.5 ± 7.9	130.0 ± 7.2	0.769	0.445
Diastolic blood pressure (mmHg)	83.0 ± 6.6	82.5 ± 7.2	0.280	0.780

### Comparison of antipsychotic treatment type and lactate levels

There was a significant main effect of both antipsychotic treatment types and treatment time on blood lactate levels (Table 2), demonstrating that the increase in blood lactate levels was achieved over time and more with haloperidol than with olanzapine. There was also a significant interaction between the combination of antipsychotic treatment type and treatment time on blood lactate levels (*F*(1.43, 41.6) = 8.54, *P* < 0.01). For patients in the haloperidol group, the treatment resulted in the progressive increase in blood lactate levels from baseline to each time period; for patients in the olanzapine group, although the treatment resulted in higher blood lactate levels from baseline to each time period, that difference was lower than that found in the haloperidol group (Table 3).

**Table 2:** Interaction between antipsychotic treatment type (haloperidol and olanzapine) and time on blood lactate level

	F	Within group *P* value*	Interaction *P* value*	η^2†^	α^‡^
Antipsychotic type	39.23	<0.001		0.575	1.000
Treatment time	12.69	<0.001		0.746	1.000
Antipsychotic type and treatment time	8.54		0.002	0.603	1.000

*P significance level.

†Effect size.

‡Observed statistical power.

**Table 3:** Change in blood lactate levels in patients receiving haloperidol or olanzapine antipsychotics

Month	Within-group change from baseline				Between-group difference	
	haloperidol treatment (n = 30)		olanzapine treatment (n = 30)			
	mean ± standard deviation	P*	mean ± standard deviation	P*	t	P*
0	1.41 ± 0.44		1.20 ± 0.34		2.048	0.045
1	2.21 ± 1.05	<0.001	1.21 ± 0.31	0.743	4.989	<0.001
3	2.93 ± 2.01	<0.001	1.32 ± 0.35	0.015	4.303	<0.001
6	2.51 ± 0.78	<0.001	1.39 ± 0.37	0.001	7.01	<0.001

*Two-way repeated measures ANOVA with post-hoc comparisons.

For within-group differences, post-hoc analysis by pairwise *t* test (Table 3) showed significant increase in mean blood lactate levels from baseline to each time point in the haloperidol group. In the olanzapine group, mean blood lactate levels increased from baseline to month 3 and 6, while no significant changes were found from baseline to month 1.

For between-group differences (Table 3), mean blood lactate levels between patients in the haloperidol or olanzapine group did not differ at baseline time point, but were significantly higher in patients receiving haloperidol than those receiving olanzapine at all other time points.

### Frequency of extrapyramidal side effects

The frequencies of the four extrapyramidal side effects, as scored on the ESRS, are listed in Table 4. About 27% of patients receiving haloperidol and nearly 7% of patients receiving olanzapine reported moderate symptoms of parkinsonism. Of patients receiving haloperidol, 17% did not report any symptoms and more than one-third (37%) reported symptoms of dystonia after 6 months. None of the patients receiving olanzapine reported any symptoms of dystonia or tardive dyskinesia.

**Table 4:** Extrapyramidal side effects in patients taking haloperidol or olanzapine antipsychotics

	No. (%)of patients receiving treatment with							
Symptom	haloperidol (n = 30)				olanzapine (n = 30)			
	baseline	month 1	month 3	month 6	baseline	month 1	month 3	month 6
Parkinsonism:								
absent	30 (100)	16 (53)	14 (47)	15 (50)	28 (93)	26 (87)	23 (77)	21 (70)
mild		7 (23)	7 (23)	7 (23)	2 (7)	4 (13)	5 (17)	7 (23)
moderate		3 (10)	6 (20)	8 (27)			2 (7)	2 (7)
severe		4 (13)	3 (10)					
Akathisia:								
absent	29 (97)	26 (87)	24 (80)	25 (83)	30 (100)	30 (100)	27 (90)	27 (90)
mild		2 (7)	3 (10)	3 (10)			3 (10)	3 (10)
moderate		2 (7)	2 (7)	2 (7)				
severe	1 (3)		1 (3)					
Dystonia:								
absent	30 (100)	21 (70)	18 (60)	19 (63)	30 (100)	30 (100)	30 (100)	30 (100)
mild		5 (17)	6 (20)	8 (27)				
moderate		3 (10)	5 (17)	3 (10)				
severe			1 (3)					
Tardive dykinesia:								
absent	30 (100)	27 (90)	26 (87)	26 (87)	30 (100)	30 (100)	30 (100)	30 (100)
mild		3 (10)	4 (13)	2 (7)				
moderate				2 (7)				
severe								

### Correlation between blood lactate and extrapyramidal side effects

There was a strong and significant positive correlation between blood lactate and parkinsonism at month 1 (r = 0.64, *P* < 0.001) and moderate and significant positive correlation at month 6 (r = 0.44, *P* = 0.02) in patients receiving haloperidol (Table 5). Also, there was a strong and significant positive correlation between blood lactate and dystonia (r = 0.62, *P* < 0.001), and moderate and significant positive correlation between blood lactate and tardive dyskinesia at month 1 (r = 0.39, *P* = 0.03).

**Table 5:** Correlation between lactate and extrapyramidal side effects in patients receiving haloperidol treatment over time (n = 30)

Side effects	Blood lactate			
	baseline	month 1	month 3	month 6
	r (*P*)	r (*P*)	r (*P*)	r (*P*)
Parkinsonism:				
baseline	0*			
month 1		0.64 (<0.01)		
month 3			0.31 (0.092)	
month 6				0.44 (0.016)
Akathisia:				
baseline	-0.03 (0.865)			
month 1		-0.15 (0.424)		
month 3			-0.23 (0.226)	
month 6				-0.26 (0.16)
Dystonia:				
baseline	0			
month 1		0.62 (<0.01)		
month 3			0.22 (0.251)	
month 6				0.45 (0.012)
Tardive dyskinesia:				
baseline	0			
month 1		0.39 (0.032)		
month 3			0.30 (0.113)	
month 6				0.31 (0.102)

*Symptom reported as absent in patients.

In patients receiving olanzapine, there was a correlation between blood lactate and parkinsonism at baseline and 3 and 6 months (Table 6). There was no correlation between blood lactate and akathisia, dystonia, or tardive dyskinesia at any time points (Table 5, Table 6).

**Table 6:** Correlation between lactate and extrapyramidal side effects in patients receiving olanzapine treatment over time (n = 30)

Side effects	Blood lactate			
	baseline	month 1	month 3	month 6
	r (*P*)	r (*P*)	r (*P*)	r (*P*)
Parkinsonism:				
baseline	0.44 (0.016)			
month 1		0.36 (0.054)		
month 3			0.42 (0.019)	
month 6				0.42 (0.021)
Akathisia:				
baseline	0*			
month 1		0		
month 3			-0.09 (0.64)	
month 6				0.10 (0.611)
Dystonia:				
baseline	0			
month 1		0		
month 3			0	
month 6				0
Tardive dyskinesia:				
baseline	0			
month 1		0		
month 3			0	
month 6				0

*Symptom reported as absent in patients.

## Discussion

To our knowledge, the present study is the first that demonstrated that haloperidol or olanzapine antipsychotic therapy changed blood lactate levels and caused extrapyramidal side effects. We found significant increases in blood lactate levels and extrapyramidal side effects in both treatment groups, although the lactate levels were significantly higher in the haloperidol than in olanzapine group. Moreover, the majority of patients in the haloperidol treatment group reported extrapyramidal side effects. Our findings are consistent with previous studies on greater incidence of extrapyramidal side effects in patients treated with haloperidol, which increases the generalizability of our study. For example, several studies reported that haloperidol use was associated with increased extrapyramidal signs, particularly parkinsonism (12,36-39). Olanzapine use was associated with low incidence of extrapyramidal side effects in our study, which is also in accordance with other studies (32,39,40).

Previous studies have found hyperprolactinemia in critically ill patients, but not in psychiatric patients treated with antipsychotics (13,14,30,33). These results suggest that olanzapine and other second-generation antipsychotic agents may have a lower tendency to increase blood lactate levels and cause extrapyramidal side effects over a 6-month period than first-generation agents, such as haloperidol. Although the mechanism of action of antipsychotic medications is not entirely explained, first-generation antipsychotics have antidopaminergic activity, thus inducing prolactin secretion (12).

There are several limitations of this study. First, due to the non-randomized, observational study design, we had no control over the antipsychotic assignment, and therefore direct comparisons of treatment group outcomes may be misleading. Second, there was no true control group, because it would be impractical and unethical to deny treatment to patients who need psychiatric medication. Further prospective clinical studies are needed to determine the time course and magnitude of developing higher lactate blood levels (>4.0 mmol/L) and extrapyramidal side effects.

This study showed that haloperidol therapy increases the lactate levels and the risk of parkinsonism, which affects the patients’ quality of life. Consequently, alternative antipsychotics can be prescribed with lower risk of increasing blood lactate levels (2,32,41).
